# The effects of C5-substituted 2,4-diaminoquinazolines on selected transcript expression in spinal muscular atrophy cells

**DOI:** 10.1371/journal.pone.0180657

**Published:** 2017-06-29

**Authors:** Cinsley Gentillon, Andrew J. Connell, Ryan W. Kirk, Matthew E. R. Butchbach

**Affiliations:** 1 Center for Applied Clinical Genomics, Nemours Biomedical Research, Nemours Alfred I. duPont Hospital for Children, Wilmington, Delaware, United States of America; 2 Department of Biological Sciences, University of Delaware, Newark, Delaware, United States of America; 3 Center for Pediatric Research, Nemours Biomedical Research, Nemours Alfred I. duPont Hospital for Children, Wilmington, Delaware, United States of America; 4 Department of Pediatrics, Thomas Jefferson University, Philadelphia, Pennsylvania, United States of America; Iowa State University, UNITED STATES

## Abstract

C5-substituted 2,4-diaminoquinazolines (2,4-DAQs) ameliorate disease severity in SMA mice. It is uncertain, however, that these compounds increase SMN protein levels *in vivo* even though they were identified as activators of the *SMN2* promoter. These compounds also regulate the expression of other transcripts in neuroblastoma cells. In this study, we investigate the mechanism by which the 2,4-DAQs regulate the expression of *SMN2* as well as other targets. D156844, D158872, D157161 and D157495 (RG3039) increased *SMN2* promoter-driven reporter gene activity by at least 3-fold in NSC-34 cells. These compounds, however, did not significantly increase *SMN2* mRNA levels in type II SMA fibroblasts nor in NSC-34 cells, although there was a trend for these compounds increasing SMN protein in SMA fibroblasts. The number of SMN-containing gems was increased in SMA fibroblasts in response to 2,4-DAQ treatment in a dose-dependent manner. *ATOH7* mRNA levels were significantly lower in type II SMA fibroblasts. 2,4-DAQs significantly increased *ATOH7*, *DRNT1* and *DRTN2* transcript levels in type II SMA fibroblasts and restored *ATOH7* levels to those observed in healthy fibroblasts. These compounds also increase *Atoh7* mRNA expression in NSC-34 cells. In conclusion, 2,4-DAQs regulate *SMN2* by increasing protein levels and gem localization. They also increase *ATOH7*, *DRNT1* and *DRNT2* transcript levels. This study reveals that the protective effects of 2,4-DAQs in SMA may be independent of *SMN2* gene regulation. These compounds could be used in concert with a proven *SMN2* inducer to develop a multi-faceted approach to treating SMA.

## Introduction

Proximal spinal muscular atrophy (SMA) is an early-onset neurodegenerative disease characterized by the loss of α-motor neurons in the anterior horn of the spinal cord which leads to muscle weakness and atrophy [1;2]. SMA is an autosomal recessive disease that is a leading genetic cause of infant death worldwide with an incidence of 1 in ~10,000 births [3;4]. SMA can be classified into five clinical grades based on age of onset and the highest achieved motor milestone. Regardless of clinical grade, proximal SMA results from the loss or mutation of *SMN1* (*survival motor neuron 1*) on chromosome 5q13 [5]. SMN protein is involved the assembly of small nuclear ribonucleoprotein (snRNP) complexes required for splicing [2]. SMN is also implicated in stabilizing messenger ribonucleoprotein (mRNP) complexes in axons [6]. In addition to these well characterized functions, SMN is involved in many other intracellular processes [7].

In humans a large tandem chromosomal duplication has lead to a second copy of this gene known as *SMN2* [8;9]. *SMN2* is functionally distinguishable from *SMN1* by a single nucleotide difference (*SMN2 c*.*850C>T*) in exon 7 that disrupts an exonic splice enhancer. As a result, most *SMN2* mRNAs lack exon 7 (*SMNΔ7*) and produce a protein that is both unstable and less than fully functional [10–12]. Although only 10–20% of the *SMN2* gene product is fully functional [8;9], increased genomic copies of *SMN2* inversely correlates with disease severity among individuals with SMA [13]. Studies using transgenic mouse models for SMA have shown that increased *SMN2* copy number lessens the phenotypic severity of disease [14–16]. *SMN2* copy number variation, therefore, is a major modifier of disease severity in SMA.

As *SMN2* is a major genetic modifier of SMA phenotype, it has become the primary target for the development of therapies—both small molecular and biological—for SMA. Numerous studies have identified many classes of compounds as inducers of *SMN2* expression [17]. Small molecule drugs can increase *SMN2* expression *in vivo* at different levels of gene regulation, such as enhancing promoter activity or increasing the inclusion of exon 7 in *SMN2* mRNA transcripts. C5-substituted 2,4-diaminoquinazolines (2,4-DAQs) are potent inducers of *SMN2* promoter activity that were identified through a high-throughput drug screen [18]. The piperidine derivative D156844 increases SMN expression in type II SMA fibroblasts and ameliorates the survival and phenotype of SMNΔ7 SMA mice [19–22]. RG3039, also known as D157495, increases the mean lifespan in multiple mouse models of SMA [23;24].

The 2,4-DAQs bind to and inhibit the activity of the mRNA decapping enzyme DcpS [25]. DcpS is a member of the histidine triad family of nucleotide hydrolases and is implicated in mRNA degradation [26]. After complete 3’ to 5’ degradation of the mRNA in eukaryotes by exonucleases, DcpS hydrolyzes the residual cap structure, 7-methylguanosine nucleoside triphosphate (m7GpppN), to release 5’ diphosphate-oligonucleotide mRNA and 7-methylguanosine monophosphate (m^7^GMP) as products [26;27]. DcpS is also implicated in the 5’ to 3’ mRNA decay pathway where it is found to dephosphorylate the m^7^GDP decapping product to m^7^GMP [28]. It is unclear how DcpS inhibition by the 2,4-DAQs increases *SMN2* expression. In this study, we examined the effects of a novel group of 2,4-DAQs on the regulation of *SMN2* expression in type II SMA fibroblast lines as well as in motor neuron-based reporter cell lines. We also examined the effects of these 2,4-DAQs on the expression of three DcpS-regulated transcripts [29]—*ATOH7* (*atonal homologue 7*), *DRNT1* (*DcpS-responsive noncoding transcript 1*) and *DRNT2*—in type II SMA fibroblasts.

## Materials and methods

### Drug compounds

The C5-substituted 2,4-diaminoquinazoline derivatives D156844, D157495 (RG3039), D157161 and D158872 were obtained from Repligen Corp. (Waltham, MA) and were all dissolved in DMSO.

### Cell culture

Fibroblasts derived from type II SMA (GM03813, GM22592 and AIDHC-SP22) and non-SMA (GM03814, AIDHC-NMC1, AIDHC-SC1 and AIDHC-SC2) individuals were grown in DMEM containing 10% EquaFETAL (Atlas Biologicals, Fort Collins, CO), 2 mM L-glutamine (Life Technologies, Grand Island, NY) and 1% penicillin-streptomycin (Life Technologies). GM03813 [30], GM22592 and GM03814 [30] fibroblast lines were obtained from Coriell Cell Repositories (Camden, NJ) while the other fibroblast lines were generated at Nemours/Alfred I. duPont Hospital for Children. All type II SMA fibroblast lines used in this study contain 0 copies of *SMN1* and 3 copies of *SMN2* [31]. GM03814 fibroblasts [30] were derived from the carrier mother of GM03813 and contain 1 copy of *SMN1* and 5 copies of *SMN2* [31]. The other non-SMA fibroblast lines contain 2 copies of *SMN1* and 2 copies of *SMN2* [31]. The fibroblast lines were authenticated using short tandem repeat profiling and digital PCR as described previously [32].

The mouse motor neuron cell line NSC-34 [33] and the NSC-34-based reporter lines [18;34] were maintained in DMEM, 5% EquaFETAL, 2 mM L-glutamine and 1% penicillin/streptomycin. In all instances, the cells were maintained in a humidified chamber at 37°C and 5% CO_2_.

### β-Lactamase reporter assays

The clone 11 cell line (Vertex Pharmaceuticals, [18]) was used for the *SMN2* promoter assay and the clone 5.3 (Vertex Pharmaceuticals, [34]) was used for the *SMN2* splicing assay. The cells were seeded onto a black-walled, clear bottom 96-well tissue culture plates (Santa Cruz Biotechnology) at a density of 5×10^4^ cells/well. For these experiments, the compounds were tested in quadruplicate. Drug compounds were added to the medium using a 96-pin replicator (pin diameter = 1.19 mm; V&P Scientific, Inc., San Diego, CA) and plates were incubated for 19 hours. At the end of the incubation, 20μL of 6X CCF2-AM dye (GeneBlazer In Vivo Detection Kit, Life Technologies) was added to each of the assay wells and plates were incubated at room temperature for 2 hours before the plates are read on a plate reader (Victor X4, Perkin Elmer). Fluorescence intensities (F) were obtained at 2 wavelengths for each sample: the CCF2-AM substrate at λ_em_ = 530 nm and the cleaved product at λ_em_ = 460 nm. The normalized fluorescence intensity ratio (F_460_:F_530_) was plotted against compound concentration and used to generate a dose response curve for the *SMN2* promoter assay.

### Treatment of cells with 2,4-DAQs

All cells were plated 24 hours prior to treatment with drug compounds and harvested by scraping following five days of treatment. This treatment paradigm was selected to monitor changes in response to chronic exposure to these compounds, which would be similar to conditions observed in SMA patients treated with one of these compounds. Medium was changed daily and fresh drug compounds or DMSO was added at a 1:1000 dilution every 24 ± 2 hours during the five-day treatment period.

### Immunofluorescence and gem count analysis

For immunofluorescence, cells were seeded onto gelatinized glass coverslips at a density of 4000 cells/cm^2^ and treated with compounds as described above. Immunostaining of fibroblast cells was accomplished as described previously [19;35] using the MANSMA2 mouse anti-SMN mAb (1:200; Developmental Studies Hybridoma Bank, Iowa City, IA [36]). SMN immunostaining within the nuclei of treated fibroblasts was visualized using a DMRXA2 epifluorescence microscope (Leica Microsystems) with an ORCA-ER cooled camera (Hamamatsu, Hamamatsu City, Japan) and Volocity 6.1.1 software (Perkin-Elmer). Gems were counted 10 randomly selected nuclei in a field of view; this process was repeated for a total of 10 randomly selected, non-overlapping fields of view. The following parameters were measured: the number of gems, the number of cells with gems and the number of cells with more than 1 gem.

### Quantitative RT-PCR

Cells were seeded onto 6-well plates at a density of 3.2×10^4^ cells/well and treated as described above. The total RNA was extracted from cell lines using the RNAeasy Mini columns (QIAGEN, Germantown, MD), according to the manufacturer’s recommendations. First-strand complementary DNA was carried out using the iScript cDNA synthesis kit (Bio-Rad, Hercules, CA) according to manufacturer’s directions. Quantitative PCR was performed in a 384 well plate on a 7900HT Fast Real-Time PCR system (Applied Biosystems, Foster City, CA). Target transcripts were amplified by real time polymerase chain reaction using the SYBR Green PCR Master Mix (QIAGEN) in 10 μL total volume and the following cycling conditions: a 10-minute initial denaturation step at 95°C, followed by 40 cycles of 15 seconds at 95°C and 1 minute at 60°C. All samples were assayed in triplicate. The following primer sets (Integrated DNA Technologies, Coralville, IA) were used: *ATOH7* [29], (F) 5’-AAAGCTGTCCAAGTACGAGAC-3’, (R) 5’-CGAAGTGCTCACAGTGGAG-3’; *DRNT1* [29], (F) 5’-CACCTAGACTCATCACTTAGATCCACC-3’, (R) 5’-GAGACCTGATGGCTACAACTGACA-3’; *DRNT2* [29], (F) 5’-TGGAGAAGCGATGGATGACAGAGA-3’, (R) 5’-GGTGAACGGACACAATTGCCAGAA-3’; *PAQR8* [29], (F) 5’-AACGTCTGGACCCATTTACTG-3’, (R) 5’-CAGGTGAGGTAAGTGATTGAC-3’; *SMNex6Fq*, 5’-CCATATGTCCAGATTCTCTTGATGA-3’; *SMNex78Rq*, 5’-ATGCCAGCATTTCTCCTTAATTTA-3’; *SMNex68Rq*, 5’-ATGCCAGCATTTCCATATAATAGC-3’; *Smn*, (F) 5’-AGAATGCCACAACTCCCTTG-3’, (R) 5’-ATCCAGTATAAACCACGACACAG-3’ and *Atoh7*, (F) 5’-CAAGCTCTCCAAGTACGAGAC-3’, (R) 5’-TCTACCTGGAGCCTAGCAC-3’. For the fibroblast samples, data for each transcript were normalized to the geometric mean of three reference transcripts, *ACTB* (*β-actin*), *GAPD* (*glyceraldehyde 3-phosphate dehydrogenase*), and *RPLP0* (*ribosomal protein lateral stalk subunit P0*), to minimize the variability in the expression of a single reference [37]. The data for each NSC-34 sample were normalized to the geometric mean of *Rpl13a* (*ribosomal protein L13a*) and *Pgk* (*phosphoglycerate kinase*). The reference primer sets were obtained from RealTime Primers LLC (Elkins Park, PA). The relative transcript levels were calculated using the efficiency-adjusted 2^-ΔΔCt^ method [38;39]. The PCR efficiency (E) for each primer set was calculated from the slope of a Ct vs. log10(cDNA serial dilution) curve (E = 10^[-1/slope]^) [40]. ΔC_t,adjusted_ is the difference between the adjusted C_t_ (C_t,measured_ × E) for the target transcript and the geometric mean of the adjusted C_t_ values for the three reference genes and ΔΔC_t_ is defined as the difference between the ΔC_t_ for the SMA sample and the ΔC_t_ for the control sample.

### mRNA stability assay

mRNA stability was assayed as described previously with modification [41]. Fibroblasts were seeded onto 6-well plates at a density of 3.2×10^4^ cells/well and treated with compounds or DMSO as previously described. Twenty-four hours after the final treatment, cells were exposed to 5 μg/mL actinomycin D (ActD; Sigma-Aldrich, St. Louis, MO) for 0, 2, 6, 12 or 24 hours. RNA isolation and first strand complementary DNA synthesis were performed as already described. The thermocycling profile was 50°C for 2 minutes, 95°C for 10 minutes, followed by 30 cycles at 95°C for 15 seconds and 60°C for 1 minute, and a final cycle at 72°C for 5 minutes. PCR was performed using primers specific for sequences within *SMN* exons 6 and 8: *SMN* exon 6 (F), 5’-CCCATATGTCCAGATTCTCTTGAT-3’; *SMN* exon 8 (R), 5’-CTACAACACCCTTCTCACAG-3’. *COL3A (collagen IIIA*) was used as control because of its high expression in fibroblasts [41]: COL3A (F), 5’-GCTCTGCTTCATCCCACTATT-3’; COL3A (R), 5’-GGAATACCAGGGTCACCATTT-3’. The PCR products were electrophoresed through a 2% agarose gel. Gel images were captured with an AlphaImager system (ProteinSimple, San Jose, CA) and band intensities were quantified using AlphaView, version 3.2.2.

### Immunoblot

For protein analysis, cells were plated onto 10-cm dishes at a density of 4.0×10^5^ cells/dish and treated with test compounds as previously described. The resultant cell pellets were lysed in 50 μL cell lysis buffer (0.1% Triton X-100 and Complete protease inhibitor cocktail (Roche Life Sciences, Indianapolis, IN) in phosphate-buffered saline (PBS, pH 7.4)). Lysates were sonicated using a Sonic Dismembrator (Thermo Scientific). Protein quantification was performed using the Micro BCA Protein Assay kit (Fisher Scientific). NSC-34 (5 μg/lane) or 10 μg fibroblast (10 μg/lane) extracts were mixed with 0.2-volumes non-reducing 6× loading dye (10.28% SDS, 36% glycerol and 0.012% bromophenol blue in 350 mM Tris-HCl, pH 6.8) and 0.1-volumes 1 M DTT, were heated at 90°C-100°C for 10 minutes and briefly centrifuged. Prepared samples along with Kaleidoscope Precision Plus protein ladder (Bio-Rad) were resolved through 12% Polyacrylamide Mini-PROTEAN TGX Precast gels (Bio-Rad) using the Mini-PROTEAN system (Bio-Rad). Samples were then transferred onto polyvinylidine fluoride (PVDF) membranes (0.45 μm, GE Healthcare Life Sciences, Piscataway, NJ) using the Trans-Blot semi dry transfer cell (Bio-Rad) and incubated for 1 h at room temperature in 1×blocking buffer (PBS containing 5% milk and 0.1% Tween-20). Blots were then incubated in primary antibody solution (in 0.2×blocking buffer) overnight at 4°C. The following primary antibodies were used: mouse anti-SMN mAb (MANSMA2 (8F7), Developmental Studies Hybridoma Bank, Iowa City, IA [36], 1:100), rabbit anti-ATH5 (ATOH7) pAb (EMD Millipore; 1:200), rabbit anti-MATH5 (ATOH7) pAb (Abcam; 1:100), rabbit anti-ATOH7 pAb (Thermo Scientific; 1:1000), rabbit anti-MATH5 (ATOH7) mAb (EPR13935, Abcam; 1:1000), mouse anti-β-actin mAb (AC-15, Sigma-Aldrich, 1:5000) and mouse anti-β-tubulin mAb (E7, Developmental Studies Hybridoma Bank [42], 1:100). The blots were extensively washed with PBS containing 0.1% Tween-20 (PBST; 3×10 min) and incubated for 1 h at room temperature with an HRP-linked anti-mouse or anti-rabbit IgG secondary antibody (1:5000; Rockland Immunochemicals, Inc., Pottstown, PA) diluted in 0.2×blocking buffer. After extensive washing, the bound antibody was detected by chemiluminescence using either the Western Sure ECL Substrate (LiCor, Lincoln, NE) or SuperSignal West Femto (Thermo Scientific) kits and developed with the C-DiGit Blot Scanner (LiCor). Band intensities, defined as the band signal divided by the band area, were measured using the Image Studio^TM^ Lite software (LiCor). The measured band areas were the same for each sample on a blot. Band intensities for the target protein (SMN) were divided by those for the reference protein (β-actin or β-tubulin) to obtain normalized band intensities. To measure the relative protein level for a sample, the normalized band intensity for the drug-treated sample was divided by the normalized band intensity for the control sample (either DMSO-treated cells or a reference cell line).

### Data and statistical analysis

Data are expressed as mean ± standard error. Parametric data were analyzed by ANOVA with either a Bonferroni (gem analysis) or Holm-Sidak (expression analysis) *post hoc* test. Statistical significance was set at p ≤ 0.05. Comparisons between data were performed with Sigma Plot v.12.0 or SPSS v.22.0. The EC_50_ value of each compound for the *SMN2* promoter assay was determined using Prism (GraphPad).

## Results

### Effects of C5-substituted 2,4-DAQs on SMN-containing gem localization in type II SMA fibroblasts

**Fig 1:**
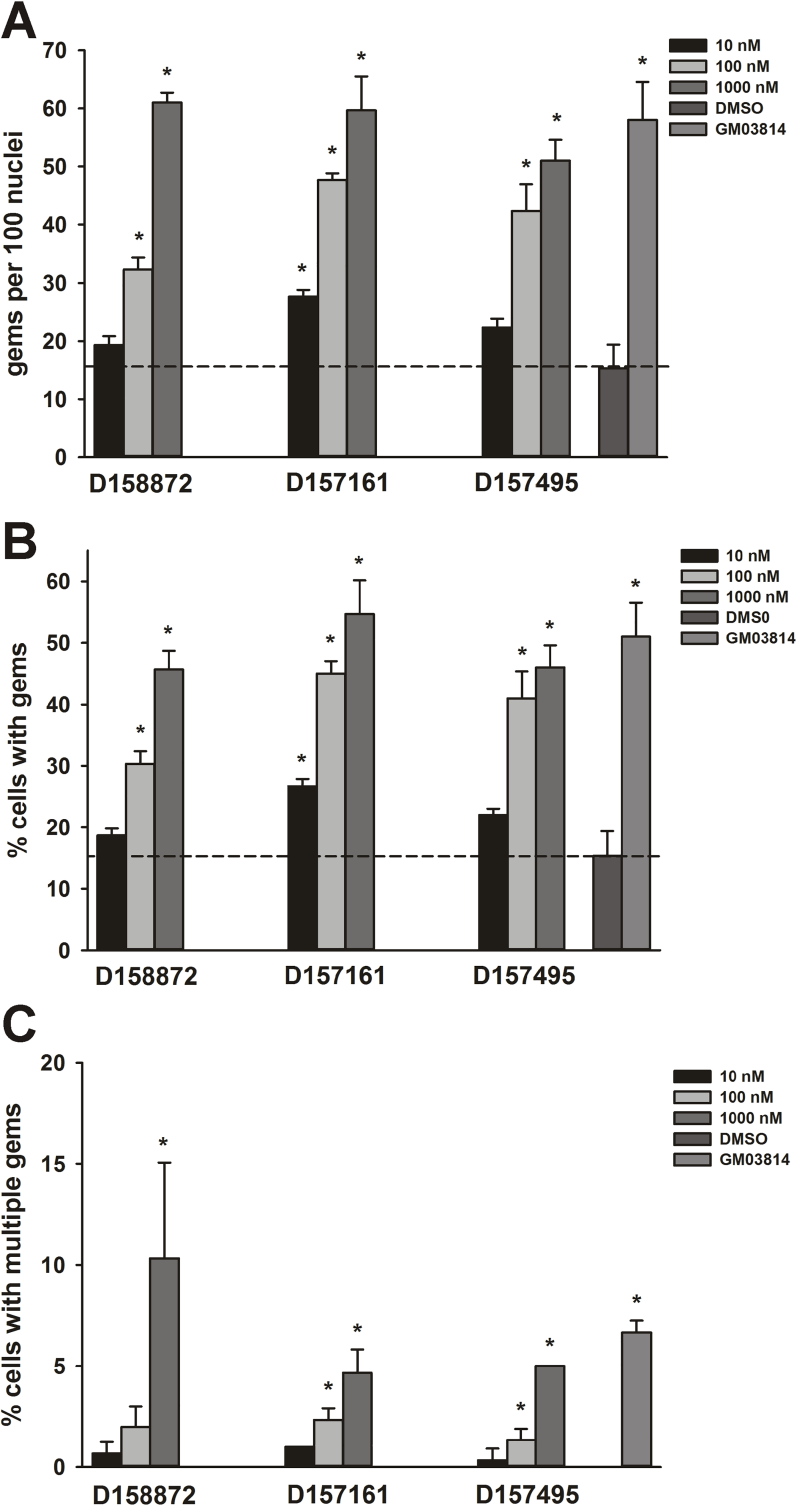
Effects of C5-substituted 2,4-DAQs on SMN localization to the nucleus in GM03813 type II SMA fibroblasts. Cells were treated with different doses (10–1000 nM; n = 3/dose/drug) of D158872, D157161, D157495 or DMSO (vehicle) for 5 days. The number of SMN-positive gems within 100 randomly selected nuclei was counted. As a control, the number of gems in healthy, carrier fibroblasts (GM03814) was also measured. The gem count analysis was expressed as *(A)* the number of gems per 100 nuclei, *(B)* the proportion of cells containing gems and *(C)* the proportion of cells containing multiple gems. The dashed line represents the value for DMSO-treated GM03813 fibroblasts. The asterisk (*) denotes a statistically significant (p ≤ 0.05) difference between drug- and vehicle-treated cells.

In most cells, SMN localizes to discreet foci within the nucleus known as gems [43]. In SMA cells, the number of subnuclear gems is greatly reduced and the magnitude of gem deficit is related to clinical severity [44]. As the 2,4-DAQ D156844 has been previously shown to increase the number of SMN-positive gems within the nuclei of SMA cells [19], we examined the effects of three other C5-substituted 2,4-DAQs—D158872, D157161 and D157495—on gem localization in GM03813 type II SMA fibroblasts. Each compound increased the number of gems (Fig 1A) in SMA fibroblasts in a dose-dependent manner. The proportion of fibroblasts containing at least one subnuclear gem (Fig 1B) and more than one gem (Fig 1C) also increased in a dose-dependent manner. At the highest doses tested (1 μM), each compound increased the gem counts to those observed in healthy, carrier GM03814 fibroblasts.

### Effects of C5-substituted 2,4-DAQs on *SMN2* gene regulation

**Fig 2:**
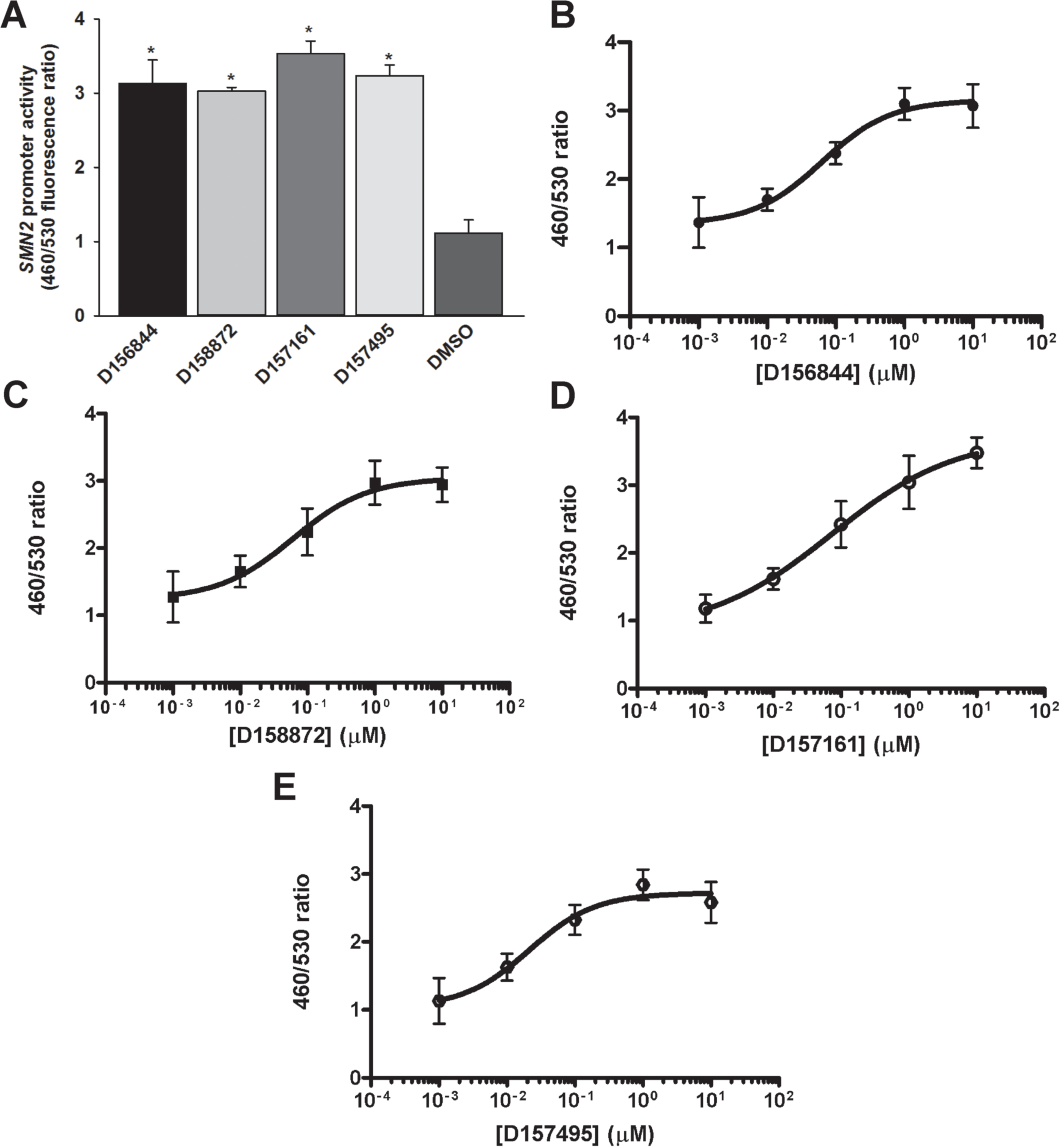
Effects of C5-substituted 2,4-DAQs on *SMN2*-drived BLA activity. Clone 11 NSC-34 cells harboring a reporter gene driven by the 3.4-kb *SMN2* promoter were treated with 1 μM D156844, D158872, D157161, D157495 or DMSO (n = 4/drug) for 19 hours prior to fluorescent β-lactamase assay analysis. *(A)* All 4 compounds significantly increased *SMN2*-drived BLA activity. The asterisk (*) denotes a statistically significant (p ≤ 0.05) difference between drug- and vehicle-treated cells. Dose-response curves (1 nM– 10 μM) for D156844 *(B)*, D158872 *(C)*, D157161 *(D)* and D157495 *(E)*. Each compound tested exhibited a dose-dependent increase in *SMN2*-drived BLA activity.

We first examined the effects of C5-substituted 2,4-DAQs on *SMN2* promoter activity using clone 11 cells. This NSC-34 clonal line contains a β-lactamase (BLA) reporter gene under the control of a 3.4-kb fragment of the *SMN2* promoter; this clonal line has been previously used in high-throughput screening of *SMN2* inducers [18]. When compared to DMSO (vehicle)-treated cells, D156844, D158872, D157161 and D157495 significantly increased *SMN2*-drived BLA activity as measured by an increase in the λ_460_:λ_530_ fluorescence emission ratio (Fig 2A). All four compounds displayed sigmoidal dose-dependent increases in *SMN2*-drived BLA activity (Fig 2B–2E). Based on EC_50_ values, D157495 was 2.5-fold more potent at increasing *SMN2*-drived BLA activity than D156844 (Table 1). These compounds were ranked as follows based on their potencies at inducing *SMN2*-drived BLA activity: D157495 > D156844 = D158872 > D156171.

**Table 1:** EC_50_s of the C5-substituted 2,4-DAQs on *SMN2*-drived BLA activity.

compound	mean EC_50_ (nM)	R^2^
D156844	58.36 ± 0.0075	0.9923
D158872	54.59 ± 0.017	0.9991
D157161	77.17 ± 0.014	0.0991
D157495	23.30 ± 0.0042	0.9744

The effects of the C5-substituted 2,4-DAQs on the activity of BLA driven by the 3.4-kb *SMN2* promoter were measured in clone 11 reporter cells. Each calculated EC_50_ was expressed as the mean ± SEM (n = 4).

**Fig 3:**
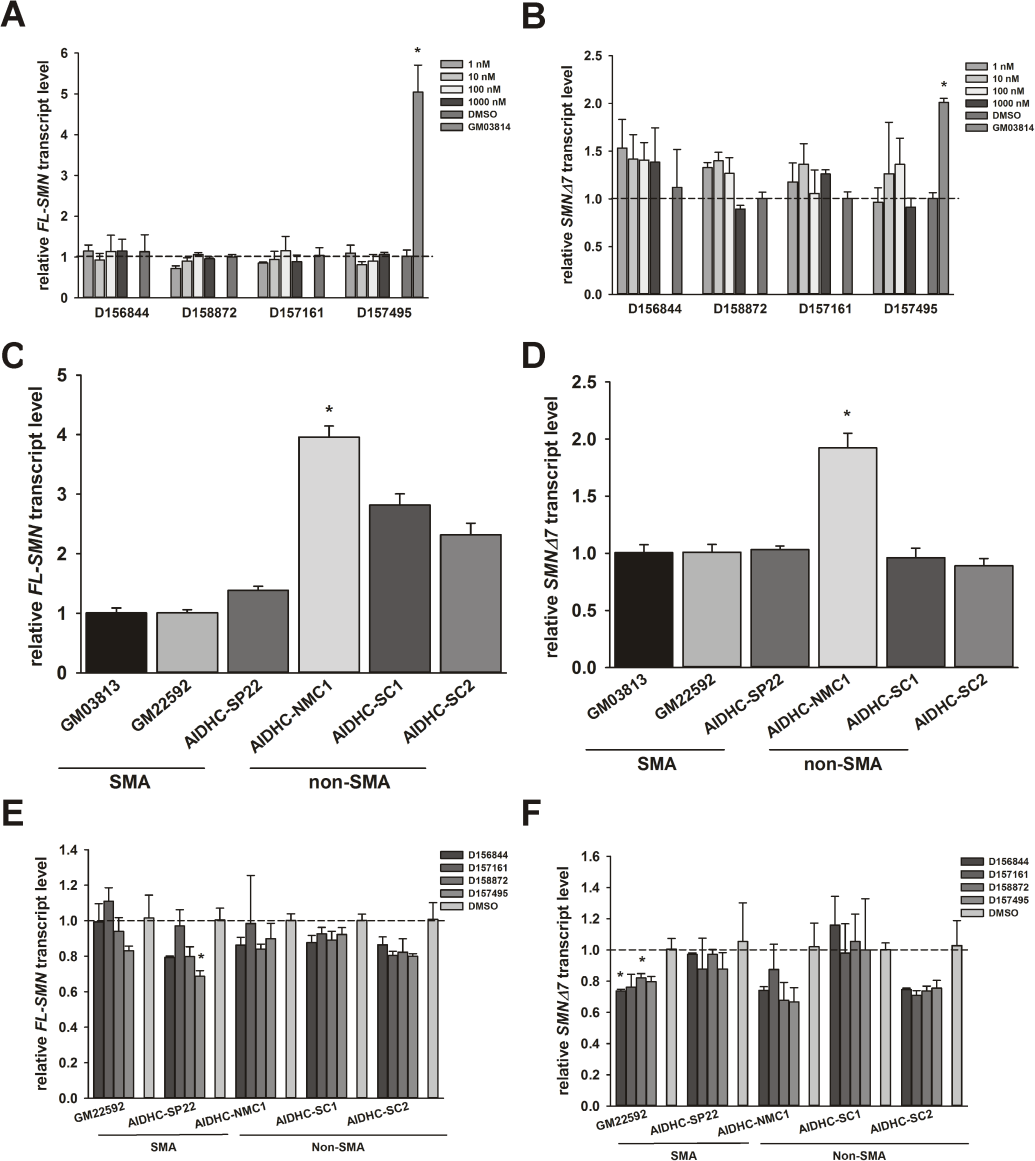
Effects of C5-substituted 2,4-DAQs on expression of *full-length SMN* (*FL-SMN*) and *SMNΔ7* mRNA levels in fibroblasts. Fibroblasts were treated with 1–1000 nM D156844, D158872, D157161, D157495 or DMSO for 5 days. mRNA levels of *FL-SMN* and *SMNΔ7* were measured via quantitative RT-PCR with *ACTB*, *GAPD* and *RPLP0* being used as reference transcripts. The levels of either *FL-SMN (A)* or *SMNΔ7 (B)* mRNAs were not affected by the compounds in GM03813 fibroblasts. All transcript levels were expressed relative to DMSO-treated, GM03813 cells (dashed line). The basal levels of *FL-SMN (C)* and *SMNΔ7 (D)* mRNAs were measured in 3 different type II SMA (GM03813, GM22592 and AIDHC-SP22) and non-SMA (AIDHC-NMC1, AIDHC-SC1 and AIDHC-SC2) fibroblast lines. All transcript levels were expressed relative to GM03813 cells (dashed line). These fibroblast lines were subsequently treated for 5 days with 1 μM D156844, D158872, D157161, D157495 or DMSO. An increase in either *FL-SMN (E)* or *SMNΔ7 (F)* mRNA levels was not observed in any cell line treated with these C5-substituted 2,4-DAQs. All transcript levels were expressed relative to DMSO-treated cells for each fibroblast line (dashed line).

The effects of C5-substituted 2,4-DAQs on *SMN* mRNA levels were examined in GM03813 fibroblasts. Quantitative RT-PCR showed that the amounts of full-length SMN (*FL-SMN*) (Fig 3A) and exon 7 lacking SMN (*SMNΔ7*) (Fig 3B) did not increase in response to treatment with D156844, D158872, D157161 or D157495. To determine whether or not these observations were unique to this fibroblast line, we also examined the effects of the C5-substituted 2,4-DAQs on *SMN* mRNA levels in two additional type II SMA fibroblast lines, GM22592 and AIDHC-SP22, that possess the same *SMN2* copy number as GM03813 cells [31]. As a control, *FL-SMN* and *SMNΔ7* mRNA levels in response to D156844, D157161, D158872 and D157495 were measured in three healthy fibroblast lines—AIDHC-NMC1, AIDHC-SC1 and AIDHC-SC2—that have 2 copies of *SMN1* and 2 copies of *SMN2* [31]. Under basal conditions, *FL-SMN* (Fig 3C) mRNA levels were lower in type II SMA fibroblast lines when compared against healthy fibroblasts. With the exception of AIDHC-NMC1, basal *SMNΔ7* (Fig 3D) mRNA levels were not different between type II SMA and healthy fibroblasts. *FL-SMN* (Fig 3E) or *SMNΔ7* (Fig 3F) mRNA levels were not affected by the compounds in either SMA or healthy cells. The variability in responsiveness to these compounds was observed in both SMA and healthy fibroblasts.

**Fig 4:**
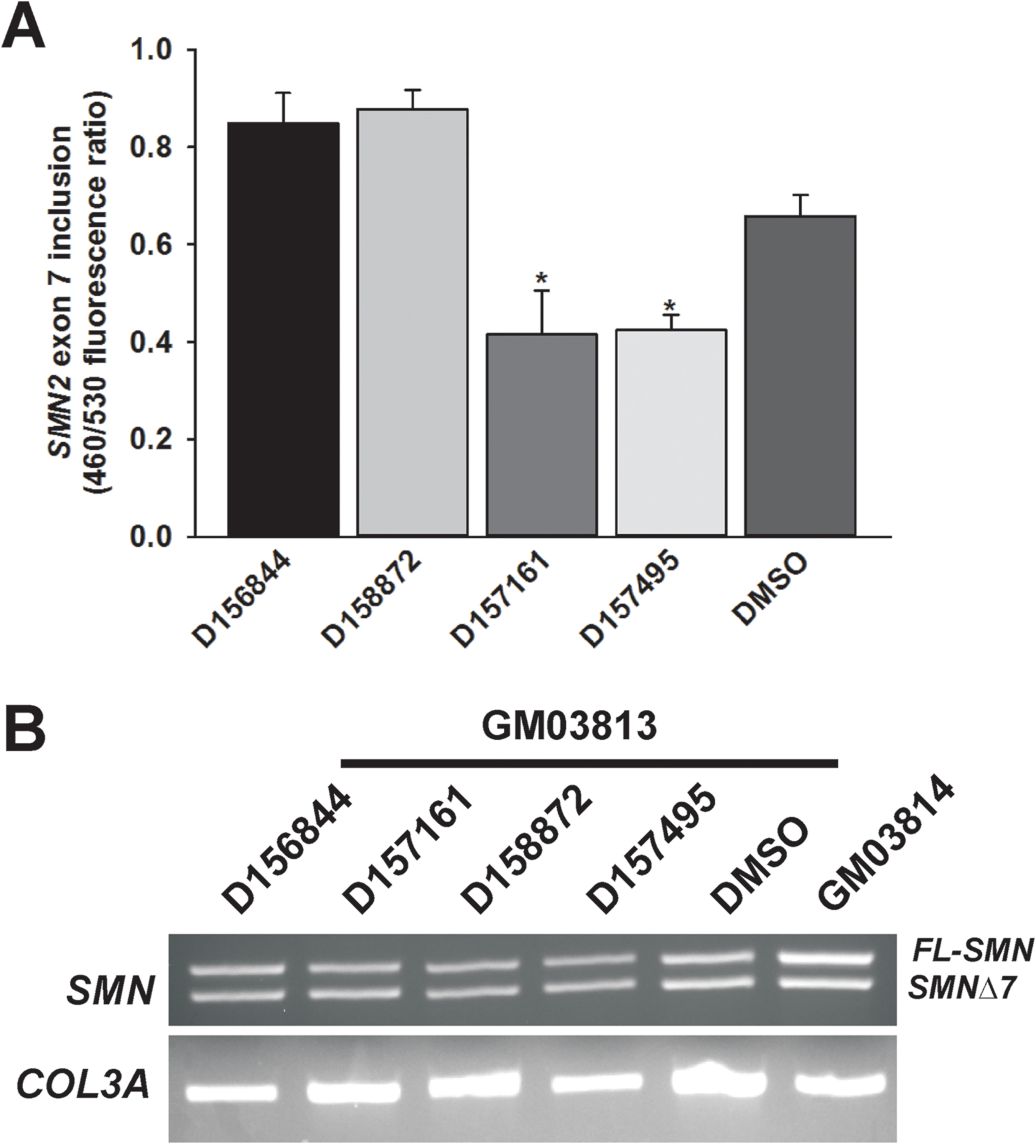
Effects of C5-substituted 2,4-DAQs on alternative splicing of *SMN2* mRNAs. *(A)* Clone 5.3 NSC-34 cells harbor a reporter gene whose expression is linked to inclusion of *SMN2* exon 7. These cells were treated with 1 μM D156844, D158872, D157161, D157495 or DMSO (n = 4/drug) for 19 hours prior to fluorescent β-lactamase assay analysis. These compounds tested did not increase the inclusion of *SMN2* exon 7 but D157161 and D157495 actually decreased *SMN2* exon 7 inclusion. The asterisk (*) denotes a statistically significant (p ≤ 0.05) difference between drug- and vehicle-treated cells. *(B)* Qualitative analysis of the effects of D156844, D157161, D158872 and D157495 on *SMN2* transcripts in type II SMA fibroblasts. GM03813 cells were treated with 1 μM each compound or DMSO (n = 3/compound) for 5 days and then analyzed for changes in the amounts of *FL-SMN* and *SMNΔ7* transcripts by RT-PCR and agarose gel electrophoresis. The amounts of *FL-SMN* and *SMNΔ7* transcripts were also compared against GM03814 samples. *COL3A* transcripts were also assayed as a loading control for RT-PCR. These compounds tested did not affect the proportion of *FL-SMN* relative to *SMNΔ7*.

In addition to examining the effects of C5-substituted 2,4-DAQs on *FL-SMN* and *SMNΔ7* mRNA levels, we also measured changes in the alternative splicing of *SMN2* mRNAs at exon 7. Clone 5.3 NSC-34 cells contain a BLA reporter gene whose expression is dependent on inclusion of exon 7 in the SMN mini-gene construct [34]. Clone 5.3 cells were treated with 1 μM D156844, D158872, D157161 and D157495 and then assayed for *SMN2* exon 7 inclusion, i.e. increased BLA activity. *SMN2* exon 7 inclusion was not altered by these compounds (Fig 4A); in fact, two of the compounds, D157161 and D157495, reduced *SMN2* exon 7 inclusion. The effects of these compounds on the splicing of exon 7 were also measured in type II SMA fibroblasts. GM03813 cells were treated with 1 μM D156844, D157161, D158872, D157495 or DMSO for 5 days; *FL-SMN* and *SMNΔ7* transcripts were measured by qualitative RT-PCR and agarose electrophoresis. The proportion of *FL-SMN* or *SMNΔ7* mRNAs in these cells were not affected by these compounds (Fig 4B).

**Fig 5:**
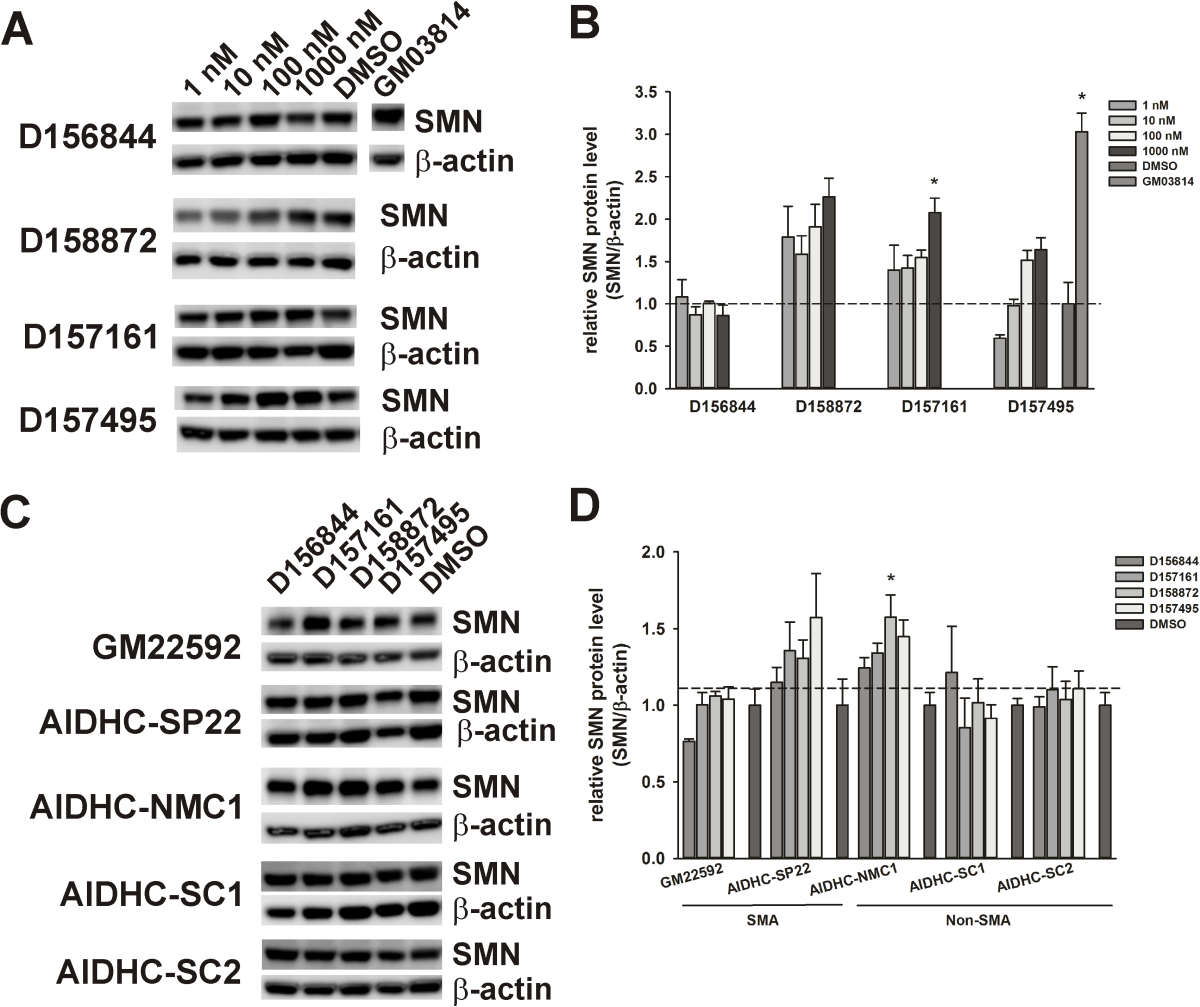
Effects of C5-substituted 2,4-DAQs on SMN protein levels in fibroblasts. *(A)* Representative SMN and β-actin immunoblots of GM03813 type II SMA fibroblasts treated for 5 days with 1–1000 nM D156844, D158872, D157161, D157495 or DMSO. *(B)* Relative SMN protein levels—expressed as the ratio between SMN and β-actin band intensities—in GM03813 fibroblasts treated with 2,4-DAQs. All SMN protein levels were expressed relative to DMSO-treated GM03813 fibroblasts. *(C)* Representative SMN and β-actin immunoblots of type II SMA and non-SMA fibroblasts treated for 5 days with 1 μM D156844, D158872, D157161, D157495 or DMSO. *(D)* Relative SMN protein levels in type II SMA and non-SMA fibroblasts treated with 1 μM 2,4-DAQs. All SMN protein levels were expressed relative to DMSO-treated cells for each fibroblast line (dashed line). The asterisk (*) denotes a statistically significant (p ≤ 0.05) difference between drug- and DMSO-treated cells.

The effects of C5-substituted 2,4-DAQs on *SMN* protein levels in GM03813 fibroblasts were measured using immunoblot. D158872, D157161 and D157495 increased SMN protein levels in treated GM03813 cells (Fig 5A and 5B). SMN protein levels in cells, however, were not affected by treatment with D156844. SMN protein levels were increased in GM33592 type II SMA and AIDHC-NMC1 healthy fibroblasts (Fig 5C and 5D). Some variability in responsiveness to 2,4-DAQs was observed between cell lines.

### Effects of C5-substituted 2,4-DAQs on *SMN2* mRNA stability

**Fig 6:**
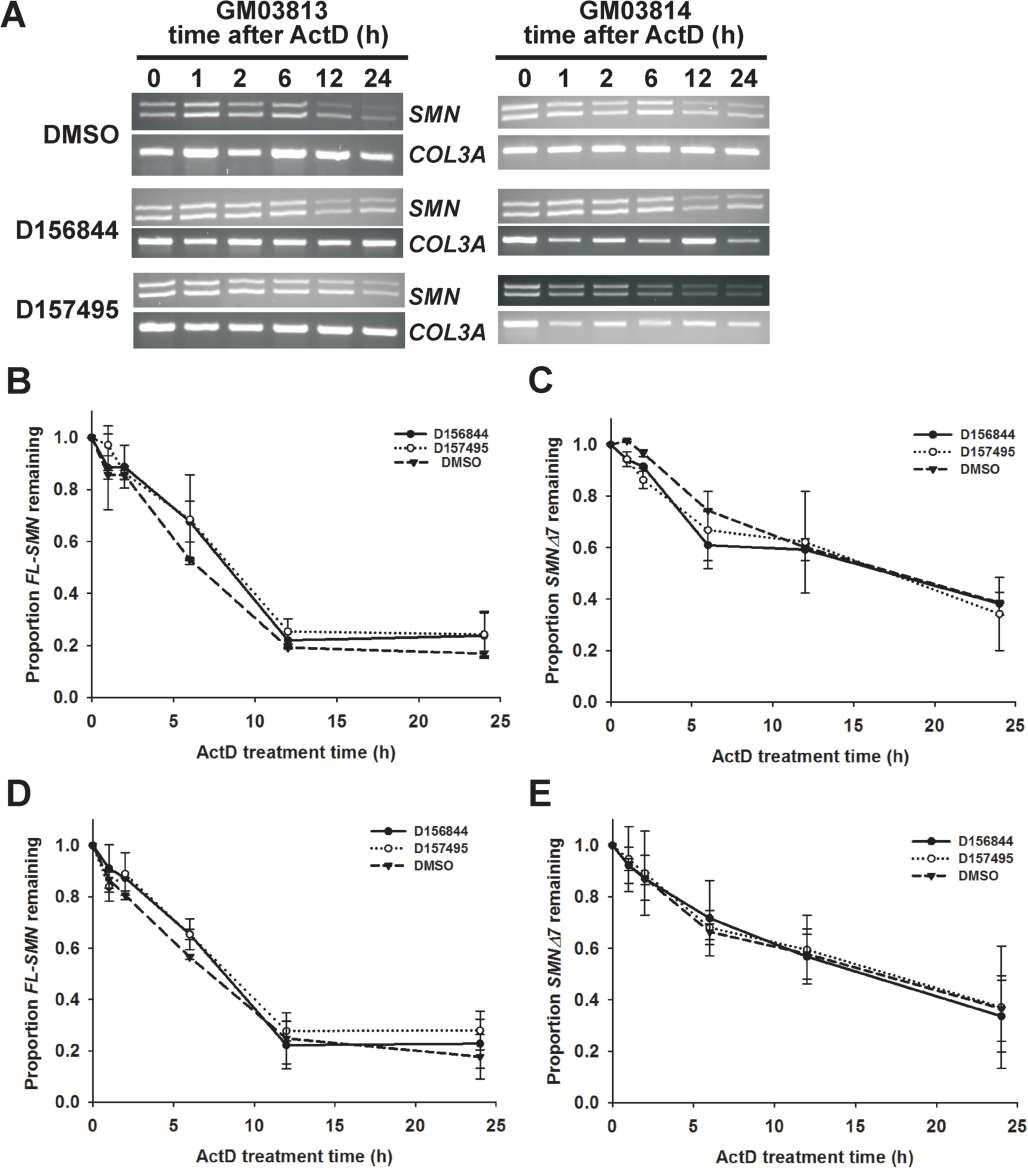
Effects of C5-substituted 2,4-DAQs on the stabilities of *FL-SMN* and *SMNΔ7* mRNAs in fibroblasts. *FL-SMN* and *SMNΔ7* mRNA stabilities were measured in fibroblasts pre-treated for 5 days with either 1 μM D156844, 1 μM D157495 or DMSO and then exposed to 5 μg/mL actinomycin D (ActD) for 0–24 hours. *FL-SMN*, *SMNΔ7* and *COL3A*—a positive control for mRNA degradation—mRNAs were detected by qualitative RT-PCR and agarose electrophoresis. *(A)* Qualitative analysis of *SMN* and *COL3A* transcript stabilities from GM03813 and GM03814 fibroblasts treated with D156844, D157495 or DMSO prior to ActD exposure. Stability of *FL-SMN (B*, *D)* and *SMNΔ7 (C*,*E)* mRNAs over time in GM03813 *(B*,*C)* or GM03814 fibroblasts treated with D156844 (solid circle and solid line), D157495 (open circle and dotted line) or DMSO (closed triangle and dashed line).

C5-substituted 2,4-DAQs act as inhibitors of the mRNA decapping enzyme DcpS [25]. Since DcpS activity is required for mRNA degradation [45], these compounds may stabilize *FL-SMN* and *SMNΔ7* transcripts thereby increasing SMN protein levels in SMA cells. To test this hypothesis, GM03813 and GM03814 fibroblasts were treated with D156844 and D157495 for 5 days and then exposed to the transcriptional inhibitor ActD for up to 24 hours. The degradation of *FL-SMN* and *SMNΔ7* mRNAs were monitored by RT-PCR and agarose gel electrophoresis; *collagen IIIA* (*COL3A*) mRNA degradation was also monitored as a control [41]. Qualitatively, D156844 and D157495 increase *FL-SMN* and *SMNΔ7* transcripts in GM03813 fibroblasts after 12 h exposure to ActD (Fig 6A). Neither treatment with D156844 or D157495, however, affected the rates of degradation for *FL-SMN* or *SMNΔ7* mRNAs either in GM03813 (Fig 6B and 6C) or in GM03814 (Fig 6D and 6E) cells. These compounds, therefore, do not affect *SMN2* mRNA stability in fibroblasts.

### Effects of C5-substituted 2,4-DAQs on DcpS-responsive transcripts in SMA fibroblasts

**Fig 7:**
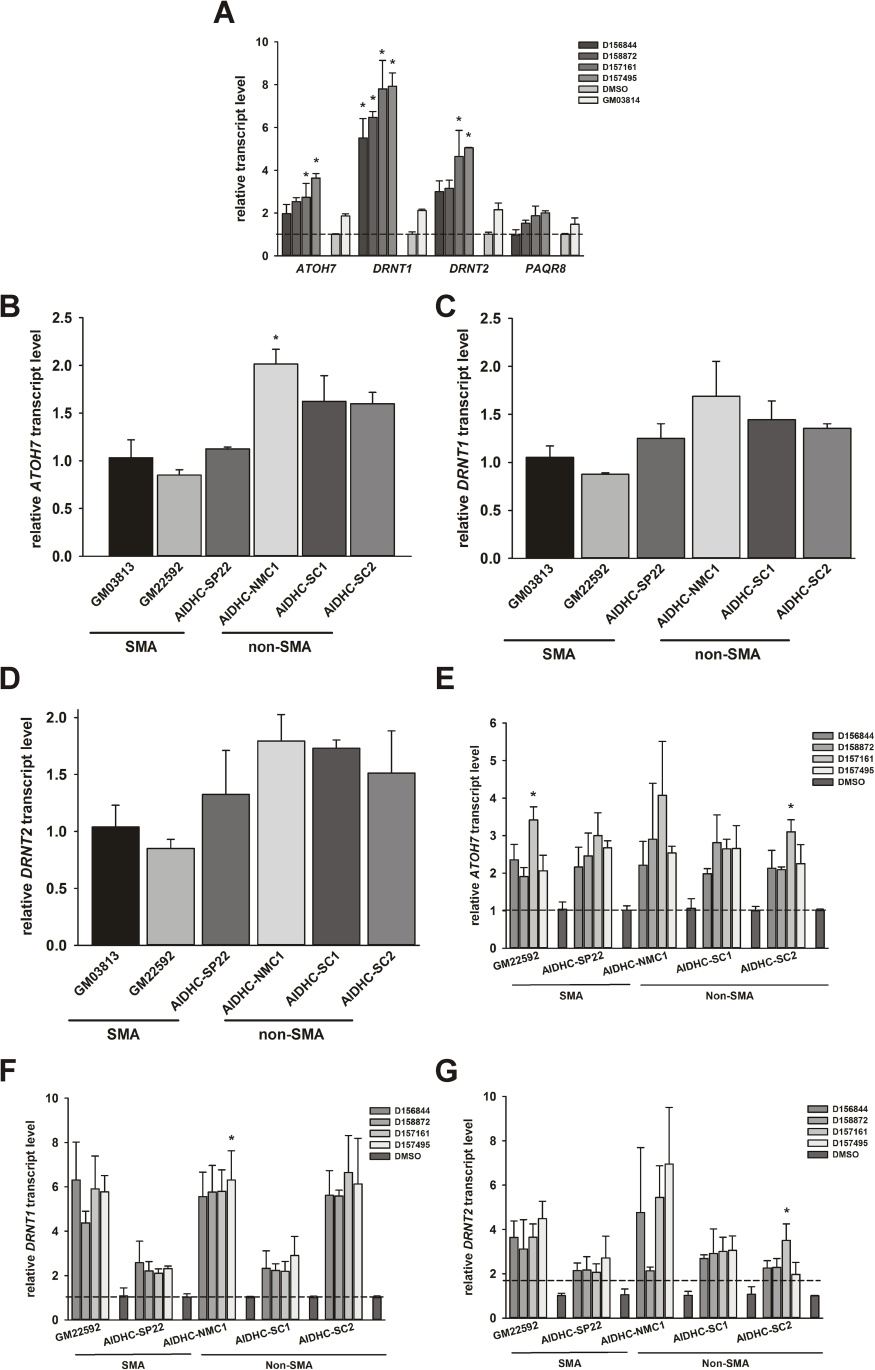
Effects of C5-substituted 2,4-DAQs on the mRNA expression of DcpS regulated transcripts. *(A)* GM03813 fibroblasts were treated with 1 μM D156844, D158872, D157161, D157495 or DMSO for 5 days. mRNA levels of *ATOH7*, *DRNT1*, *DRNT2* and *PAQR8* were measured via quantitative RT-PCR with *ACTB*, *GAPD* and *RPLP0* being used as reference transcripts. All of the 2,4-DAQs tested increased *ATOH7*, *DRNT1* and *DRNT2* transcript levels in SMA fibroblasts. All transcript levels were expressed relative to DMSO-treated, GM03813 cells (dashed line). The asterisk (*) denotes a statistically significant (p ≤ 0.05) difference between drug- and DMSO-treated cells. The basal levels of *ATOH7 (B)* mRNA as well as *DRNT1 (C)* and *DRNT2 (D)* lncRNAs were measured in 3 different type II SMA (GM03813, GM22592 and AIDHC-SP22) and non-SMA (AIDHC-NMC1, AIDHC-SC1 and AIDHC-SC2) fibroblast lines. *ATOH7* mRNA levels were higher in non-SMA fibroblasts than in SMA fibroblasts. Under basal conditions, *DRNT1* and *DRNT2* lncRNA levels are not significantly different between type II SMA and non-SMA fibroblasts. All transcript levels were expressed relative to GM03813 cells (dashed line). The asterisk (*) denotes a statistically significant (p ≤ 0.05) difference relative to GM03813 fibroblasts. These fibroblast lines were separately treated for 5 days with 1 μM D156844, D158872, D157161, D157495 or DMSO and monitored for changes in *ATOH7 (E)*, *DRNT1 (F)* and *DRNT2 (G)* transcript levels. Increases in *ATOH7*, *DRNT1* and *DRNT2* transcript levels were observed in all fibroblast lines treated with these C5-substituted 2,4-DAQs. All transcript levels were expressed relative to DMSO-treated cells for each fibroblast line (dashed line). The asterisk (*) denotes a statistically significant (p ≤ 0.05) difference between drug- and DMSO-treated cells.

We measured the effects of the 2,4-DAQs on the levels of DcpS regulated transcripts [29]—*ATOH7*, *PAQR8* (*progestin/adipoQ receptor 8*), *RAB26*, *DRNT1* and *DRNT2*—in SMA fibroblasts. All of these transcripts except for *RAB26* were expressed in fibroblasts. GM03813 fibroblasts were treated with 1 μM D156844, D158872, D156171, D157495 or DMSO for 5 days. All four compounds significantly increased *ATOH7* mRNA as well as *DRNT1* and *DRNT2* long noncoding RNA (lncRNA) levels in GM03813 SMA fibroblasts (Fig 7A).

Interestingly, the levels for each of the transcripts examined were lower in GM03813 fibroblasts when compared to healthy cells. Based on this observation, we measured *ATOH7*, *DRNT1* and *DRNT2* transcript levels in 3 different type II SMA fibroblast lines (GM03813, GM22592 and AIDHC-SP22) relative to 3 different healthy fibroblast lines (AIDHC-NMC1, AIDHC-SC1 and AIDHC-SC2). *ATOH7* mRNA levels were lower in the 3 type II SMA fibroblasts tested when compared against the 3 non-SMA fibroblasts used in this study (Fig 7B). In contrast, the levels of *DRNT1* (Fig 7C) and *DRNT2* (Fig 7D) lncRNAs were not significantly different between SMA and non-SMA fibroblasts although there was a trend for reduced levels of these transcripts in type II SMA fibroblasts. The inductive effects of all four 2,4-DAQs on *ATOH7* (Fig 7E) mRNA as well as *DRNT1* (Fig 7F) and *DRNT2* (Fig 7G) lncRNA expression were observed in both type II SMA and non-SMA fibroblasts.

The changes in ATOH7 protein levels were measured by immunoblot. Unfortunately, we could not detect a protein band with the appropriate size (17 kDa) in fibroblast samples using 4 different antibodies directed against ATOH7 (data not shown). We were able to detect bands at ~34 kDa and ~50 kDa but it was unclear if these bands were specific to ATOH7. This observation has been made in other studies involving ATOH7 protein expression [46].

### Effects of C5-substituted 2,4-DAQs on *Smn* and *Atoh7* expression in NSC-34 cells

**Fig 8:**
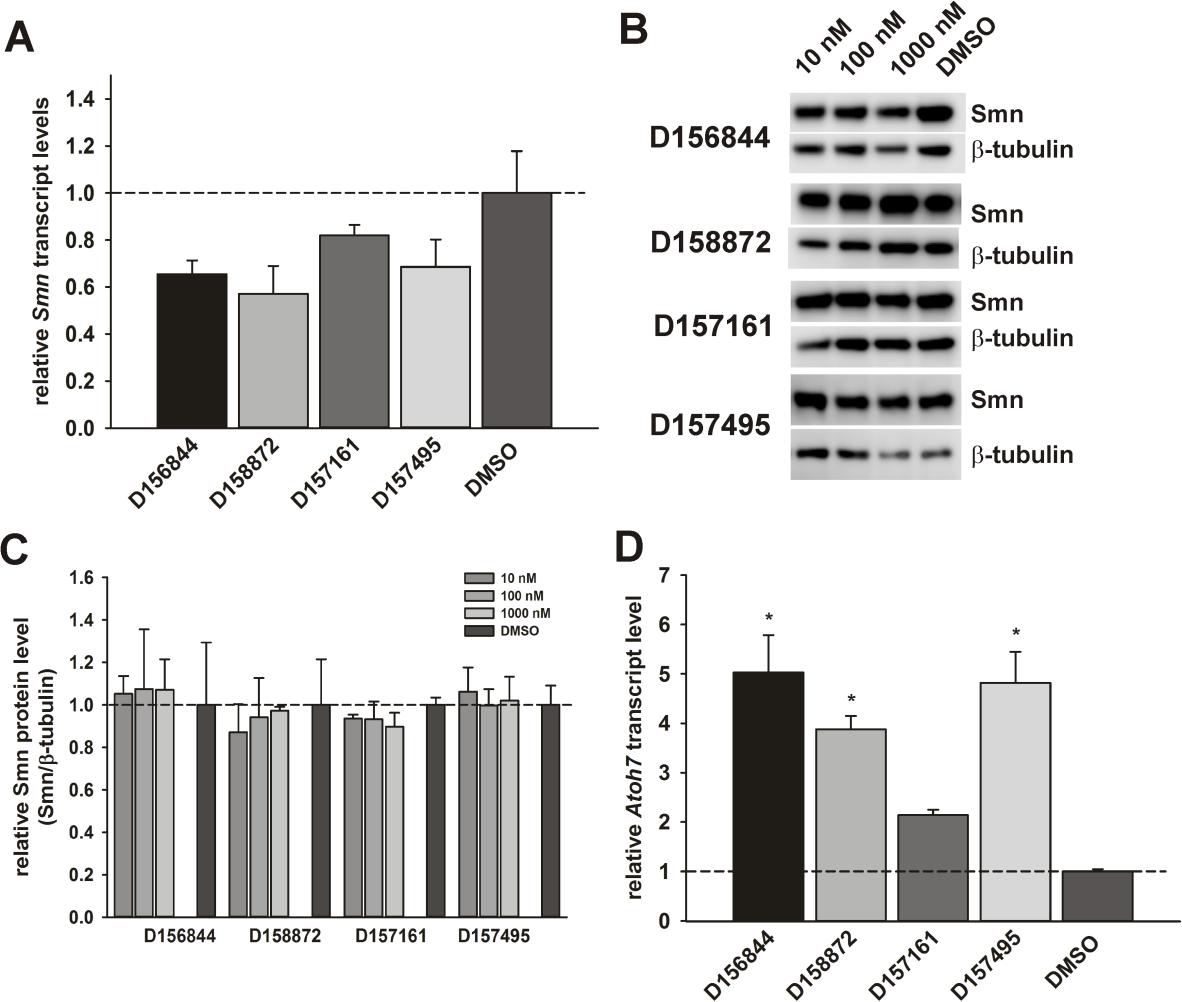
Effects of C5-substituted 2,4-DAQs on *Smn* and *Atoh7* expression in NSC-34 cells. NSC-34 cells were treated with 1 μM D156844, D158872, D157161, D157495 or DMSO for 5 days (n = 3/compound). These compounds did not affect *Smn* (*A*) mRNA levels as measured by qRT-PCR. Treated NSC-34 cells were also measured for Smn protein levels by immunoblot. (*B*) Representative SMN and β-tubulin immunoblots of NSC-34 cells treated with 1 μM D156844, D158872, D157161, D157495 or DMSO for 5 days. (*C*) Relative Smn protein levels in NSC-34 cells treated with 2,4-DAQs. Smn protein levels were not increased by these compounds in NSC-34 cells. (*D*) *Atoh7* mRNA levels were measured in treated NSC-34 cells by qRT-PCR. All 4 compounds significantly increased *Atoh7* mRNA levels in NSC-34 cells. The asterisk (*) denotes a statistically significant (p ≤ 0.05) difference between drug- and DMSO-treated cells.

The lack of effect of the 2,4-DAQs on *SMN2* gene regulation in SMA fibroblasts may be due to the cell type assayed since *SMN2* promoter activity was monitored in motor neuron-like NSC-34 cells and not in fibroblasts. To address this possibility, we examined the effects of the 2,4-DAQs on the expression of the murine SMN gene (*Smn*) in NSC-34 cells. *Smn* mRNA levels were not affected by treatment of NSC-34 cells with D156844, D158872, D157161 or D157495 for 5 days (Fig 8A). Smn protein levels were not affected by these compounds in NSC-34 cells (Fig 8B and 8C).

We also determined the effect of the 2,4-DAQs on *Atoh7* expression in a motor neuron environment. *Atoh7* mRNA levels were increased by 2.5–3.0 fold in treated NSC-34 cells (Fig 8D). Atoh7 protein levels, however, could not be measured due to the lack of a specific antibody against this antigen.

## Discussion

*SMN2* is an endogenous genetic modifier of SMA disease severity [13]. Many SMA therapeutics discovery programs aim to increase the amount of SMN protein from *SMN2* by exploring multiple mechanisms including increasing transcription from the *SMN2* promoter, enhancing the inclusion of exon 7 in the *SMN2* mRNA or stabilizing SMNΔ7 protein [17]. Initially identified from an ultrahigh throughput drug screen [18], C5-substituted 2,4-DAQs activate *SMN2* promoter activity and also increase SMN localization to subnuclear gems in SMA patient fibroblasts [19]. D156844 and D157495 (RG3039) have been shown to improve motor neuron function and extend survival of SMA mice models [20–24]. In this study, we examined the effects of four 2,4-DAQs—D156844, D158872, D157161 and D157495—on different levels of *SMN2* gene regulation. Our results show that these compounds increased reporter gene activity which is driven by a 3.4-kb fragment of the *SMN2* promoter by least 3-fold in the motor neuron-like NSC-34 cell line, with D157495 being the most potent inducer. Surprisingly, these compounds had no detectable effects on *SMN2* mRNA levels in type II SMA fibroblasts but they tended to increase SMN protein levels in these cells.

How can we explain the fact that 2,4-DAQs activate the *SMN2* promoter but fail to increase SMN2 mRNA levels? Stabilization of the β-lactamase transcripts caused by inhibition of DcpS may cause an apparent 2,4-DAQ-induced increase in promoter activity in the clone 11 cell line. This scenario, however, is unlikely because there was no increase in β-lactamase activity in clone 5.3 cells—which are used as an indicator of *SMN2* exon 7 inclusion [34]—in response to 2,4-DAQ treatment. Another possibility for these disparate results may be that the effects of the 2,4-DAQs on *SMN2* expression are specific to either cell-type or species. The promoter reporter assay is housed in NSC-34 cells, which are a fusion between mouse motor neurons and neuroblastoma [33;47], while the *SMN2* expression studies were completed in type II SMA fibroblasts. We did not observe any changes in *Smn* mRNA or protein levels in NSC-34 cells treated with 2,4-DAQs suggesting that the cell-type specificity does not explain these disparate findings. The promoter assay used in this study contains a 3.4-kb fragment of the *SMN2* promoter [18]. This promoter fragment contains all the regulatory elements that facilitate *SMN2* gene transcription [48;49]. One limitation of this *SMN2* promoter assay is that it does not take into account distal regulatory elements that regulate *SMN2* expression. As a result, a given compound, like a 2,4-DAQ, may elicit a positive response with a fragment of the *SMN2* promoter but not increase *SMN2* expression in the context of the entire *SMN2* gene.

The 2,4-DAQs did increase the number of SMN-containing gems in SMA fibroblasts in a dose-dependent manner even though these compounds did not increase *SMN2* mRNA or protein levels. These compounds may regulate the trafficking of SMN from the cytosol into the nucleus where it forms gems. This altered subnuclear localization of SMN may result from the induction of 2,4-DAQ-regulated genes such as *ATOH7*, *DRNT1* or *DRNT2*. Future work will investigate the regulation of SMN localization within the nucleus by the 2,4-DAQs and the importance of 2,4-DAQ-regulated genes in this process.

The 2,4-DAQs bind to and inhibit the activity of the human mRNA scavenger decapping enzyme DcpS [25]. This enzyme functions at multiple levels in the regulation of gene expression. DcpS cleaves the cap structures resulting from exosome degradation of mRNAs [26]. By inhibiting DcpS, the 2,4-DAQs cause the accumulation of the m7GpppN cap structure [25] which can sequester the nuclear cap binding protein and decrease the efficiency of first intron removal [50]. This results in a dysfunction in translation and the normal processing of mRNAs.

In addition to adversely affecting mRNA processing, DcpS inhibition affects the transcriptional gene regulation of certain transcripts. In SH-SY5Y neuroblastoma cells, DcpS knockout or treatment with D157495 increased the mRNA levels of *ATOH7* as well as the putative lncRNAs *DRNT1* and *DRNT2* [29]. We found that type II SMA fibroblasts have significantly lower levels of *ATOH7* than healthy fibroblasts; *DRNT1* and *DRNT2* transcript levels tended to be lower in SMA fibroblasts but the differences were not statistically significant. Interestingly, treatment of type II SMA fibroblasts with 2,4-DAQs restored *ATOH7* mRNA expression to levels observed in non-SMA fibroblasts. *ATOH7*, a single exon gene, encodes a basic helix-loop-helix (bHLH) transcription factor that is homologous to *Drosophila* proneural gene *atonal* [51]. *Atoh7*, also known as *Math5* in mice, is strongly expressed in the embryonic retina and the tenth cervical ganglion [51;52]. *Atoh7* is also expressed in other regions of the nervous system like the cochlear nucleus [53] as well as in motor neuron-like NSC-34 cells (this study). Mutations in *ATOH7* or in its promoter result in a myriad of optic disorders including optic nerve hypoplasia, persistent hyperplasia of the primary vitreous and primary open angle glaucoma [54–61]. *ATOH7* is required for optic nerve and ganglion cell development in a context-dependent manner and plays a key role in ocular embryogenesis [62–65]. Retinal neurons show deficits in neurite outgrowth in a mouse model for *Smn* deficiency (*Smn*^*2B/-*^ mice) [66]. The inhibited retinal neuritogenesis in *Smn* deficient mice may result from reduced *Atoh7* expression. Future studies will determine the importance of *Atoh7* in the development of SMA retinae. The pathogenesis of SMA may also involve the optic system, which needs to be further explored.

In summary, we found that 2,4-DAQs regulate *SMN2* expression at the protein and post-translational (i.e. gems) levels. The small increases in SMN protein resulting from 2,4-DAQ exposure in SMA fibroblasts is consistent with previous work in SMA mice wherein these compounds show a modest increase in SMN protein *in vivo* [20–24]. These compounds also increase the abundance of *ATOH7*, *DRNT1* and *DRNT2* transcripts. These *SMN2*-independent differentially expressed transcripts could be a possible neuroprotective target for SMA therapeutics. 2,4-DAQs have multiple neuroprotective effects on SMA that result from a modest increase in SMN expression as well as regulation of other neuroprotective transcripts. These compounds could be used as small molecule neuroprotectants for SMA, possibly in combination with other *SMN2* inducers, since they are CNS active and has an investigational new drug (IND) status. Examples of other *SMN2* inducers include the splice switching oligonucleotide Spinraza (nusinersen; [67–69]), which is the first FDA-approved SMA therapeutic, or the small molecule *SMN2* exon 7 splicing modifier RG7800 [70–72].
